# Early Life Adversity and Adult Social Behavior: Focus on Arginine Vasopressin and Oxytocin as Potential Mediators

**DOI:** 10.3389/fnbeh.2019.00143

**Published:** 2019-07-26

**Authors:** Nine F. Kompier, Christian Keysers, Valeria Gazzola, Paul J. Lucassen, Harmen J. Krugers

**Affiliations:** ^1^Brain Plasticity Group, Swammerdam Institute for Life Sciences, Center for Neuroscience, University of Amsterdam, Amsterdam, Netherlands; ^2^Social Brain Lab, Netherlands Institute for Neuroscience, Royal Netherlands Academy of Arts and Sciences, Amsterdam, Netherlands; ^3^Department of Psychology, University of Amsterdam, Amsterdam, Netherlands

**Keywords:** early life stress, oxytocin, vasopressin, social behavior, aggression, social recognition, social motivation

## Abstract

Exposure to stress during the early postnatal period (i.e., early life stress, ES) can impact brain physiology and modify individual variability in adult social behavior. Arginine vasopressin (AVP) and oxytocin (OXT) are two centrally released neuropeptides that are involved in shaping essential social behaviors, like aggression, social recognition, and social motivation. AVP and OXT modulate activity in brain regions important for the establishment of social behavior, and may be particularly sensitive to ES. In this review, we discuss whether ES alters the characteristics of the AVP- and OXT- systems in rodents, and whether these changes are associated with later alterations in aggression, social recognition, and social motivation. We have integrated causal studies indicating that (1) ES affects AVP/OXT, and (2) that changing AVP/OXT in affected regions alters social behavior. Although there is encouraging evidence that ES causes AVP- and OXT-system changes, and that these may mediate social behavior, a comprehensive understanding of the exact nature of AVP- and OXT changes and whether they are causal in establishing these behavioral disturbances needs further investigation. As there are indications that ES alters AVP- and OXT characteristics in humans as well, and that these may interact with adult predisposition to psychopathology with social dysfunction, future rodent studies may lay ground for a better understanding of such changes in humans. Ultimately, this may assist in developing therapeutic strategies to target ES effects on social behavior.

## Introduction

Both preclinical and clinical studies have indicated that exposure to adverse, stressful events during the early postnatal period can have a decisive impact on brain physiology and behavior later in life (Teicher et al., 2003; Nemeroff, 2004; McCrory et al., 2010). Especially parent-offspring interactions are thought to be instrumental, with dysfunctional parental care persistently affecting important processes during this period of rapid brain development, including neurogenesis and synapse formation (Teicher et al., 2003). Deviations in developmental trajectories can affect key brain structures modulating human social behavior, like the amygdala and prefrontal cortex, and thereby possibly predispose to psychiatric diseases (Teicher et al., 2003; Spinelli et al., 2009; Sarabdjitsingh et al., 2017). For example, early life adversity, like abuse and neglect, is associated with an increased risk to develop depression (Lizardi et al., 1995; Edwards et al., 2003; Heim and Binder, 2012); schizophrenia (Read et al., 2005; Morgan and Fisher, 2007); anti-social behavior (Farrington, 2005; Widom and Brzustowicz, 2006) and borderline personality disorder (Bandelow et al., 2005). Although diverse, these psychopathologies share a social component, prompting the suggestion that early adversity hampers the development of appropriate human social behaviors, and may result in dysfunction, ranging from excessive aggression to diminished social cognition and social motivation (Baribeau and Anagnostou, 2015; Tabbaa et al., 2017).

Despite evidence that the early environment plays a key role in shaping inter-individual differences in social behavior, an explanatory model linking early changes at the molecular level to complex human behavior remains elusive (Sandi and Haller, 2015). Unraveling the effects of early postnatal stress (ES) is important to understand environmentally induced individual variability in social behavior, the risk for pathology with social dysfunction, and potential targets for interference. Experimentally assessing changes after ES in humans is not feasible, as induction of ES and obtaining a variety of biological read-outs, require invasive procedures. Studies in rodents can be informative to assess behavioral and molecular effects of ES in an experimental context. Social behavior is frequently studied in rats, mice and voles, as they have evolved behavior that allows and facilitates group living (Lacey and Solomon, 2003). Using rodents as a model, ES can be induced experimentally and effects on social behavior and underlying molecular mechanisms can be revealed.

Among the suggested potential mediators that translate ES into changes in social behavior are monoamines, various neurotransmitters, corticotropin releasing hormone, cell adhesion molecules, and (neuro)peptides (Sandi and Haller, 2015). Two well-known peptides that are considered strong candidates are AVP and OXT (Veenema, 2009, 2012). Both AVP and OXT exert conserved function by mediating social behavior in mammalian species (Veenema and Neumann, 2008) and alterations in AVP and OXT in humans are associated with social dysfunction that is aggravated by ES: both are linked to social dysfunction in autism (Al-Ayadhi, 2005; Andari et al., 2010), schizophrenia (Goldman et al., 2008; Averbeck et al., 2012), depression (Heuser et al., 1998; Anderberg and Uvnäs-Moberg, 2000), and personality disorders (Coccaro et al., 1998; Lee et al., 2009). Similarly, rodent models of autism and depression frequently report changes in AVP and OXT systems (Wotjak et al., 1998; Grippo et al., 2007; Fujiwara et al., 2016). Another indirect indication for a potential role of AVP and OXT in the chain of events linking ES to changes in social behavior is the sensitivity of the AVP- and OXT-neuropeptide systems in early life. In both primates and rodents, these peptides, which are produced in specific hypothalamic nuclei and in extrahypothalamic regions, undergo transient and experience-dependent developmental changes early in life (Bales and Perkeybile, 2012; Hammock, 2014). AVP is detected as early as 11 weeks after gestation in humans (Sinding et al., 1980). OXT is detected slightly later, around 14 weeks after gestation in humans (Sinding et al., 1980). Similarly, in rodents, AVP is detected earlier than OXT, usually around gestation day 14 (AVP) and 18 (OXT) (Wolf et al., 1984; Reppert and Uhl, 1987; Altstein and Gainer, 1988; Laurent et al., 1989; Bloch et al., 1990; Farina Lipari et al., 2001). AVP- and OXT-producing cells continue to undergo rapid postnatal development in humans and rodents (Sinding et al., 1980; Wolf et al., 1984; Almazan et al., 1989). For example, in the first weeks of human life, AVP/OXT ratios change over time. In the rat, AVP- and OXT- producing cells in the MeA and BNST appear in the early postnatal days (Hammock, 2014), and receptor expression and distribution patterns undergo continuous development in this early period (Lukas et al., 2010; Hammock, 2014). Timing and abundance of these changes make them potentially sensitive to the effects of ES.

Given the similarities in AVP- and OXT-systems between rodents and humans (Box 1), we here review the current state of evidence for occurrence of changes in AVP, OXT, and their respective receptors, after ES, and whether these changes mediate social behavioral outcomes in rodents. To this end, we focus on three types of social behaviors: aggression, social recognition and social motivation (Box 2). These behaviors are at the very basis of more complex social behavior, and are often affected in psychiatric disorders that are associated with both ES and AVP/OXT (Blair, 2001; Blanchard et al., 2001; Robb, 2010; Soyka, 2011; Chevallier et al., 2012; Skuse et al., 2014) and can be and have been reliably assessed in rodents (Box 2) (Thompson et al., 2004; Kosfeld et al., 2005; Savaskan et al., 2008; Veenema and Neumann, 2008; Meyer-Lindenberg et al., 2011; Kelly and Goodson, 2014). Specifically, AVP in rodents has been found to generally promote inter-male aggression (Caldwell and Albers, 2004; Veenema et al., 2010; Terranova et al., 2017), facilitate social recognition (Bielsky et al., 2004, 2005; Veenema et al., 2012) and modulate social motivation and affiliation (Landgraf et al., 2003; Rigney et al., 2019). OXT also promotes social recognition (Ross and Young, 2009; Dumais et al., 2016; Oettl et al., 2016; Takayanagi et al., 2017), modulates social motivation and affiliation (Lukas et al., 2011b; Borland et al., 2019), and is an important regulator of maternal behavior (Olazábal and Young, 2006; Sabihi et al., 2014) and social learning (Choe et al., 2015; Nardou et al., 2019). These studies overlap with those on human behavior, which indicate that AVP and OXT are involved in regulating the same types of behaviors (Thompson et al., 2004; Kosfeld et al., 2005; Savaskan et al., 2008).

**BOX 1. AVP and OXT systems in (human) primates and rodents.**

***Synthesis and release***

Arginine vasopressin (AVP) and oxytocin (OXT) are synthesized predominantly by large magnocellular neurons whose cell bodies are situated in the hypothalamic paraventricular (PVN) and supraoptic (SON) nuclei, and in smaller, accessory nuclei between the PVN and SON (Buijs, 1987; Ludwig and Leng, 2006; Stoop, 2012). Axons of the magnocellular neurons project to the posterior pituitary gland, where action potentials trigger release into the systemic circulation (Gimpl and Fahrenholz, 2001; Baribeau and Anagnostou, 2015; Leng et al., 2015). AVP and OXT are also released centrally which is thought to be the primary way in which it influences social behavior. In humans and rodents, magnocellular neurons project to targets including the central amygdala (CeA), nucleus of the solitary tract, intermediolateral nucleus of the spinal cord, and dorsal motor nucleus of the vagus (Sofroniew et al., 1981). In addition to production in magnocellular neurons, parvocellular neurons of the PVN produce AVP and OXT. In rodents, these project centrally to the olfactory system, prefrontal cortex, entorhinal cortex, CeA and medial amygdala (MeA), bed nucleus of the stria terminalis (BNST), hippocampus, lateral septum (LS) and spinal cord (Stoop, 2012). This correlates well with primate parvocellular projection areas, which include the LS, MeA, and ventral hippocampus (Sofroniew et al., 1981). Additionally, several extrahypothalamic regions express AVP and OXT. In rodents the MeA, BNST, locus coeruleus, nucleus of the solitary tract and dorsal horn express AVP, and this largely overlaps with OXT expression (Benarroch, 2013). In humans AVP and OXT are also expressed by the locus coeruleus, but expression levels are different from rodents (Jenkins et al., 1984). Similarly to rodents, the dorsal medulla and spinal cord also express AVP and OXT in humans (Jenkins et al., 1984). Following secretion, the peptides diffuse across the extracellular space and bind to their respective receptors to exert their neuro-modulatory effects.

***Receptor structure, function, and distribution***

Arginine vasopressin exert their effects on social behavior by acting via AvpR1a and AvpR1b, and OXT via OXTRs. AvpR1a and OXTR are the most abundant receptors in the central nervous system (Stoop, 2012). **In rodents**, AvpR1a can be found in the olfactory system, LS, neocortical layer IV, basal ganglia, dentate gyrus, amygdala, BNST, ventromedial hypothalamus, thalamus and nuclei of the brain stem and spinal cord (Caldwell et al., 2008; Raggenbass, 2008). OXTR expression is more localized, and most frequently found in olfactory pathways (Freeman and Young, 2016), including the olfactory bulb and downstream areas such as the CeA and MeA, BNST, and piriform cortex. In rodents, social interactions are mainly driven by olfactory systems, which is suggested to account for high OXTR expression in these regions. Moreover, OXTR is found in areas important for social behavior, such as the LS, CA1 region of the hippocampus, nucleus accumbens (NAcc) and prefrontal cortex (Freeman and Young, 2016). **In humans and primates**, the distribution of AvpR1a and OXTR in humans is difficult to assess with a similar precision, and has been reviewed in more detail elsewhere (Baribeau and Anagnostou, 2015). Summarizing primate data, it appears that AvpR1a and OXTR are abundantly expressed in the hypothalamus, as they are in rodents (Loup et al., 1991; Boccia et al., 2013; Freeman et al., 2014). Similarly to rodents, AvpR1a is diffusely expressed throughout the central nervous system, including areas important for social behavior such as the dorsolateral part of the amygdala, the LS, and the hilar region of the dentate gyrus (Thibonnier et al., 1996). Also similarly to the rodents, OXTRs have a more localized distribution, and are most prominently expressed in the basal forebrain (Loup et al., 1991). OXTR expression is also reported to be found, albeit more variably, in limbic regions (Loup et al., 1991); the amygdala (Boccia et al., 2013), basal ganglia (Loup et al., 1991) and anterior cingulate (Boccia et al., 2013). As opposed to rodents that depend primarily on olfaction for social interactions, primates generally use more visual cues. Correspondingly, OXTR in primates is expressed at higher levels in areas for visual processing, including the superior colliculus and primary visual cortex (Freeman and Young, 2016). Thus, there are both significant similarities as well as differences in the AVP and OXT systems in rodents and primates. The similarities encourage us to examine the rodent literature, but conclusions do warrant caution in translation to humans.

**BOX 2. Description of behaviors and their respective tasks in rodents.**

**Aggression:** Innate social behaviors, intended to inflict damage on others, that allow animals to defend territories, secure resources and increase the probability of procreation (Aleyasin et al., 2018). Inappropriate aggression, such as unprovoked and out of context aggression or insensitivity to non-threatening signals, is a hallmark feature of many psychiatric disorders, including personality disorders and schizophrenia (Blair, 2001; Soyka, 2011). In rodents, aggressive behavior is often characterized by the resident-intruder test (RI-test), in which a stimulus animal is placed in the home-cage of the test animal, often inducing aggressive and territorial behaviors in the test animal, such as fighting and biting (Koolhaas et al., 2013). These behaviors, usually only seen between males, are scored as a measure of aggression. Female aggression is less easily provoked, although lactating dams display protective maternal aggression. In this case, a stimulus animal is placed in the resident cage of the dam shortly after removal of the pups. This can evoke maternal aggressive behaviors (Kemble et al., 1993).

**Social recognition:** The (long-term) capacity of individuals to differentiate among familiar, previously encountered conspecifics (Ferguson et al., 2002). Social recognition depends on social memory, which in many ways, seems to be a unique type of memory. In primates, it is primarily dependent on the visual system, whereas in rodents, the olfactory system strongly underlies social recognition (Ferguson et al., 2002). Social recognition is often impaired in neuropsychiatric disorders, such as autism (Skuse et al., 2014). In rodents, tests of social recognition score exploration times of novel and familiar mice. In the most common version of this task, a stimulus animal is placed in a cage with the test animal (acquisition phase), and is removed again. After a consolidation period, the test animal is placed with the original stimulus animal and a novel stimulus animal. The time exploring the novel stimulus animal relative to the familiar animal is scored as an indication of social recognition (Lemaire, 2003).

**Social motivation:** Prioritizing and experiencing inherent reward of social interactions and the desire to maintain and enhance relationships. In many neuropsychiatric disorders there is a strong decrease in the tendency to seek out and experience reward of social interactions (Blanchard et al., 2001; Chevallier et al., 2012). In rodents, social motivation is often assessed by scoring the time an animal spends close to others or the latency to approach a conspecific. In a standard task of social motivation a stimulus animal and a test animal are placed in a neutral cage (as opposed to the home cage of the test animal used in the resident-intruder test to trigger territorial behaviors), often inducing behaviors such as approach, sniffing, and interaction (Ikeda et al., 2013), which are scored as measure of social motivation.

We evaluate whether ES changes AVP and OXT systems in brain regions that are involved in the synthesis and release of AVP and OXT, primarily the PVN and SON, and other regions with their own synthesizing populations or receptors for AVP and OXT (amongst others, the MPOA/AH, LS, and MeA) and specifically whether these changes are instrumental in behavioral changes in aggression, social recognition and social motivation later in life. The aim of this review is thus to explore whether the AVP and OXT system could be a *causal* link between ES and changes in social behavior. We pursue this aim because understanding the nature of AVP and OXT changes after ES and their association with alterations in social behavior in rodents may provide us with new targets to rescue individuals and the society that surrounds them from the debilitating consequences of ES triggered social dysfunctions.

## Do Changes in AVP and OXT Mediate Effect of ES on Social Behavior?

Studies that have experimentally manipulated ES by altering the quantity and quality of maternal care and the interactions between the dam and her pups (Table 1) and *simultaneously* measure OXT/AVP or their receptors, and social behavior (Table 2) confirm that OXT and AVP are strong candidates to mediate the social behavioral outcomes of ES.

**TABLE 1:** Early life stress (ES) manipulation models.

**ES model**	**Manipulation**	**Control condition**	**Time frame**	**Exemplar references**
Maternal separation (MS)	Daily 3-h removal of the pups from the dam	Undisturbed litters	PDN1–PDN14	Plotsky and Meaney, 1993
Limited bedding and nesting (LBN)	Dams are provided with limited amount of bedding and nesting material, inducing changes in quality of maternal care	Litters receiving standard bedding and nesting	PDN2–PDN9	Gilles et al., 1996
Paternal deprivation (PD)	Removal from father from the litter indefinitely	Litters raised under bi-parental care	>PDN0	Ahern, 2009
Postnatal isolation (PI)	Daily 3-h removal of the pups from the dam and their littermates	Undisturbed litters	PDN0–PDN13	Wei et al., 2013

*List of experimental models most commonly used to induce ES, followed by a description of the manipulation they involve, the manipulation used as control, the time frame at which the manipulation is applied, and an exemplar reference. PND, postnatal days.*

**TABLE 2:** Effect of early life adversity on social behavior, AVP, and OXT.

**References**	**Model**	**Species**	**M/F**	**Age**	**Social behavior**	**Effects on AVP and/or OXT**	
Veenema, 2009	MS	Rat	M	Juvenile	↑ Aggression	↑ AVP-mRNA in PVN and BNST	
Tsuda et al., 2011	MS	Mouse	M	Juvenile	↓ Aggression	↑ OXT-ir in PVN ↓ AVP-ir in PVN	
Veenema et al., 2007	MS	Mouse	M	Adult	↓ Aggression	↑ AVP-ir in PVN and LH No change in OXT-ir in PVN	Aggression
Veenema et al., 2007	MS	Mouse	F	Adult	↑ Maternal aggression	↓ OXT-ir in PVN No change in AVP-ir in PVN and LH	
Veenema et al., 2006	MS	Rat	M	Adult	↑ Aggression	↑ AVP-mRNA in PVN and SON ↑ AVP-ir in PVN and SON	
Wei et al., 2013	PI	Mandarin vole	M	Adult	↓ Aggression	↓ OXT-ir in PVN ↓ AVP-ir in PVN	

Cao et al., 2014	PD	Mandarin vole	M	Adult	↓ Social recognition	↓ OXTR mRNA in MeA, NAcc No change in plasma OXT	
Cao et al., 2014	PD	Mandarin vole	F	Adult	↓ Social recognition	↓ OXTR mRNA in MeA, NAcc	Social recognition
						↓ Plasma OXT	
Lukas et al., 2011a	MS	Rat	M	Adult	↓ Social recognition	↓ AVP release in the LS	

Wei et al., 2013	PI	Mandarin vole	M	Adult	↓ Social motivation	↓ OXT-ir in PVN ↓ AVP-ir in PVN	
Tsuda et al., 2011	MS	Mouse	M	Juvenile	No change in social motivation	↑ OXT-ir in PVN ↓ AVP-ir in PVN	Social motivation

*From left to right the table reports: referenced publication; the ES model used in the publication; the species, gender (M, male; F, female) and age-group tested; the type of behavior tested, followed by the effect on AVP and OXT. Arrows indicated the direction of the effect (increase: up arrow; decrease: down arrow). If a reference is repeated, it is to mention different groups of animals in the same publication. M, male; F, female; MS, maternal separation; PVN, hypothalamic paraventricular nucleus; BNST, bed nucleus of the stria terminalis; OXT-ir, oxytocin immunoreactivity; AVP-ir, arginine vasopressin immunoreactivity; LH, lateral hypothalamus; SON, hypothalamic supraoptic nucleus; PI, post-natal isolation; PD, paternal deprivation; MeA, medial amygdala; NAcc nucleus accumbens; LS, lateral septum.*

### Aggression

In six out of six experimental groups in which aggression was measured as a consequence of ES, changes in aggressive behavior have been reported (Table 2). In three of these, aggression was increased, whereas it was decreased in three others. Aggressive behaviors were increased in male juvenile and adult rats (Veenema et al., 2006; Veenema and Neumann, 2009), but decreased in juvenile and adult mice exposed to ES (Veenema et al., 2007; Tsuda et al., 2011). In male mandarin voles, ES was associated with decreased aggression (Wei et al., 2013). Finally, in lactating female mice, ES enhanced aggressive behavior (Veenema et al., 2007). Together these results suggest that the effects of ES might differ across species, gender and age group. For instance, ES increased aggression in male rats and lactating female mice, but decreased aggression in male mandarin voles and male mice. Unfortunately, these differential effects have not yet been systematically teased apart. This currently limits our understanding of the factors that determine the directionality of the effect. However, if one ignores directionality and examines whether ES disturbs social behavior, the evidence becomes very strong indeed. Changes in male aggression are almost always accompanied by changes in AVP following a predictable pattern (Table 2). That is, enhanced aggression is associated with enhanced AVP-mRNA and enhanced AVP-protein in the PVN and SON in all experimental groups showing increased aggression (Veenema et al., 2006; Veenema and Neumann, 2009). This indicates that increased synthesis of AVP potentiates aggression in ES-animals. In line with this, rodents displaying decreased aggression, most (two out of three) experimental groups show a decrease in AVP-immunoreactivity (i.e., AVP-ir) in the PVN (Tsuda et al., 2011; Wei et al., 2013). This suggests that, generally speaking, decreased AVP synthesis in ES-animals is accompanied by decreased aggressive behaviors. This pattern is very consistent with what is known about the behavioral effects of AVP, which has a pro-aggressive role in many brain regions in male rodents and humans (Ferris and Potegal, 1988; Delville et al., 1996; Coccaro et al., 1998; Caldwell and Albers, 2004). As OXT, not AVP, is a strong regulator of maternal aggression, it makes sense that enhanced maternal aggression in ES- lactating females was related to decreased OXT-ir in the PVN, but not to AVP-ir (Veenema et al., 2007). Taken together, bi-directional changes in aggression can occur after ES, and these behavioral changes seem to *correlate* with changes in AVP and OXT in a consistent manner.

There are several remaining gaps in our knowledge. First of all, it is currently unclear whether changes within synthesizing regions in the PVN and SON affect important hubs within the social networks regulating aggression, such as the AH/MPOA and MeA (see section “Causal Pathways Linking ES to Social Behavior”). Most importantly, whether ES-induced inter-hypothalamic AVP and OXT changes are *causal* in establishing alterations in male and maternal aggression, respectively, is currently speculative, as they could be a consequence of altered behavior as well, or correlated through confounding factors. It has been shown that neonatal tactile stimulation, artificial tactile stimulation during separation, seen as a form of environmental enrichment, normalizes male aggressive behavior and AVP (Wei et al., 2013), but this is not sufficient for indicating causality, as this would require direct manipulation of the AVP and OXT system and measuring behavioral consequences.

### Social Recognition

Social recognition is negatively affected in all experimental groups after ES and is associated with decreased OXTR-mRNA in the MeA in mandarin voles (Cao et al., 2014), and with blunted AVP release in the LS of male rats (Lukas et al., 2011a) (Table 2). These results are consistent with other studies measuring social recognition after ES independently of AVP or OXT (Popik et al., 1992; Ferguson et al., 2001; Bianchi et al., 2006; Kohl et al., 2015) in which ES negatively affected social recognition.

Decreased OXT-mRNA in the MeA in combination with reduced social recognition is consistent with previous studies that identified OXTR activation in the MeA as both necessary and sufficient for social recognition in rodents (Gabor et al., 2012). The LS is an important region for social recognition, and AVP in the LS has previously been identified as having a facilitating effect on social recognition (Gabor et al., 2012; Benarroch, 2013). Lukas et al. (2011a) showed blunted AVP release and deficits in social recognition after ES. Retro-dialysis of AVP into the LS normalized social recognition in ES exposed rats. This study is seminal in indicating that the AVP changes in the LS after ES trigger changes in social recognition (Lukas et al., 2011a). To the best of our knowledge, this is the only study directly manipulating AVP or OXT in ES-animals.

### Social Motivation

Despite a variety of studies indicating that social motivation is reduced as a consequence of ES (Lukkes et al., 2009; Rincón-Cortés and Sullivan, 2016), studies simultaneously measuring AVP and OXT, and social motivation, are less conclusive. ES reduced social interaction in one out of two experimental groups (Wei et al., 2013). This reduction was accompanied by a decrease in AVP and OXT protein in the PVN of adult male mandarin voles (Wei et al., 2013). Although this speculation needs to be backed by causal evidence, environmental enrichment has been shown to normalize social motivation and AVP and OXT in the PVN (Wei et al., 2013). In male adult rats, no change in social motivation was reported as a result of ES (Tsuda et al., 2011). Interestingly, AVP protein levels were reduced in the PVN in this study, while OXT-immunoreactivity (i.e., OXT-ir) was increased in these rats exposed to ES (Tsuda et al., 2011). Being a modulator of social approach and social interactions (Heinrichs et al., 2009), this increase in OXT may actually alleviate potential behavioral effects. Unfortunately, for social motivation as well, there are no reports of extrahypothalamic changes in AVP or OXT, and again, causal evidence is still lacking.

### Conclusion

Early life stress, evoked by changing dam-pup interactions, simultaneously induces changes in AVP and OXT (immunoreactivity, release), as well as in aggression and social recognition, and less consistently, social motivation. Changes in AVP and OXT are associated with these behavioral changes in a rather predictable pattern. In line with the facilitating role of AVP in aggression, ES-associated increase in AVP is associated with increased aggression, whereas decreased AVP is linked to less aggression. Similarly, decreased OXT and AVP in regions critical for social recognition (MeA and LS, respectively) are associated with impairment in social recognition. Unfortunately, for most studies causal evidence is lacking, and the exact nature of changes (mRNA, protein, or receptor-levels) in AVP and OXT are not abundantly characterized, particularly in extrahypothalamic regions.

## Causal Pathways Linking ES to Social Behavior

**FIGURE 1:**
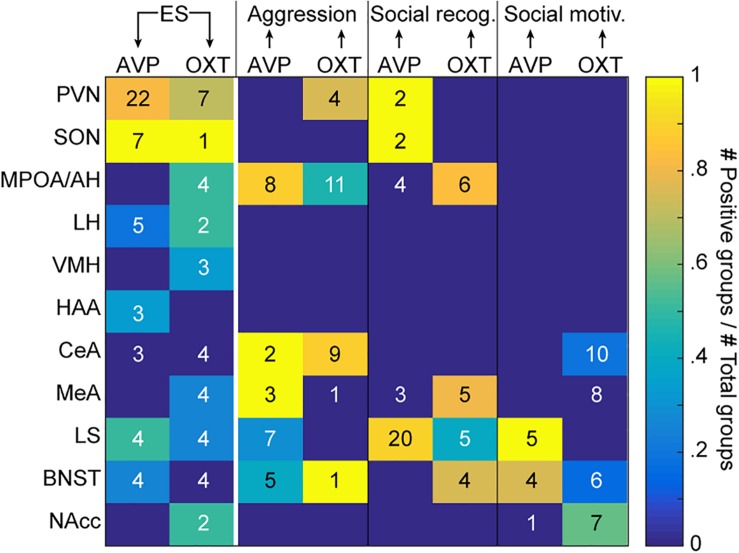
Evidence for causal relationships. The color code indicates, for each region of interest listed on the *y*-axis, the proportion of experimental groups showing a causal effect of ES on AVP or OXT (first two columns), and of AVP or OXT on the three social behaviors of interest (aggression, social recognition, and social motivation; last six columns). The number in each cell indicates the total number of tested groups. The arrows at the top of the graph indicate the directionality of the causality. Different groups can be reported together in the same manuscript (see Supplementary Tables S1, S2 for details and references). ES, early life stress; social rec, social recognition; social motiv, social motivation; PVN, hypothalamic paraventricular nucleus; SON, hypothalamic supraoptic nucleus; MPOA/AH, medial preoptic nucleus/anterior hypothalamus; LH, lateral hypothalamus; VMH, ventromedial hypothalamus; HAA, hypothalamic attack area; CeA, central amygdala; LS, lateral septum; BNST, bed nucleus of the stria terminalis.

To critically assess whether and in what brain regions the effect of ES on AVP and OXT systems could be the cause for changes in social behavior, in the text to follow we will draw on studies that focus on causality. This first includes studies that have measured the effect of ES on AVP and OXT to localize where ES alters these systems, and second, studies that have experimentally manipulated the AVP or OXT system in these brain regions to measure effects on our social behaviors of interest. Figure 1 and Supplementary Table S1 reveal a number of brain regions in which separate experiments showed (1) that ES alters the AVP and/or OXT system, and (2) that manipulating the AVP and/or OXT system in these regions leads to significant changes in at least some of the social behaviors of interest. The cited literature has been acquired by first systematically searching “early life” or “postnatal” + “stress” or “adversity” + “vasopressin” and/or “oxytocin.” For affected regions emerging from this search, we systematically searched with the key words “region” (i.e., PVN or SON or MPOA, etc.) + “vasopressin” + “oxytocin” + “aggression” or “social recognition” or “social motivation” or “social interaction.” This second search led to any study directly manipulating AVP and/or OXT within these specified regions and measuring an outcome in at least one of the behaviors of interest to be included into Figure 1 and Supplementary Table S1. Studies that do not allow for causal interference and localization of effects (for example central manipulation of AVP/OXT), or did not take place in naïve rodents (for example, rodents pre-treated with drugs) have not been included.

In addition to the main synthesizing regions (PVN and SON), we will focus on the MPOA/AH, LS and the MeA as main examples of evaluating the evidence. In terms of localization, it should be emphasized that studies so far have not systematically investigated the effects of ES on OXT and AVP systems using a whole brain approach. That a brain region is not apparent in Figure 1 thus does not reflect that it is not affected by ES.

Figure 1 reflects an attempt to integrate the studies we identified into a graphical format. The color of the cells in the two leftmost columns of the matrix reflects what proportion of studies has found a change in AVP (leftmost) and OXT (second column) in each of the regions following ES. Because some studies include multiple experimental groups, we count each group as the unit of evidence. Supplementary Tables S1, S2 contain the references of the studies included in each cell. The numerals in the cell reflect the number of groups in which the effect has been quantified in this region. As can be seen, for some brain regions like the PVN, there is broad agreement on the fact that ES alters both systems. In other areas, such as the SON, there is broad agreement for alterations of AVP, with sparser evidence for changes in OXT. For the other brain regions, the number of animal groups that have been studied is lower, and evidence is weaker for changes in OXT and AVP after ES. Except for PVN and SON, the most affected regions appear to be the LS, followed by MPOA/AH and LH, and the VMH, HAA, MeA, BNST, and NAcc. In this context, it should be noted that one of the studies that looked at the widest range of brain regions (Lukas et al., 2010, groups 024,025,026 of Supplementary Table S1) examined the effect of ES at three ages [Group 024 (5 weeks), Group 025 (8 weeks), Group 026 (16 weeks)]. In this study, and reflected in Figure 1 and Supplementary Table S1, it was found that in many brain regions, effects of ES are restricted to specific ages and one neuropeptide system.

The rest of Figure 1 then explores whether experimentally induced changes in AVP or OXT systems in each of the regions of interest has been shown to alter social behavior, using the same approach to indicate numbers of experimental groups in which the effect was tested as numerals, and proportion finding effects as color code. While the many empty cells in Figure 1 alert us to how many blind spots still exist in the experimental study of the links between ES, OXT/AVP and social behavior, we will discuss in what follows the evidence in more detail and nuance for those regions that have been more systematically explored.

### Paraventricular Nucleus and Supraoptic Nucleus

#### Causality of ES Effects on AVP and OXT in the PVN and SON

Given the large number of studies we can draw from within the PVN and SON, we will highlight what we can conclude from different parameters, or measurements at different ages or in different sexes. Results are summarized below for AVP (Table 3) and OXT (Table 4).

**TABLE 3:** AVP changes in the PVN and SON after ES.

**References**	**Model**	**Species**	**M/F**	**Age**	**AVP system changes**	
Zhang et al., 2012	MS	Rat	M	Infant	↑ AVP-mRNA in PVN and SON	
Rice et al., 2008	LBN	Mouse	M	Infant	No change in AVP-mRNA in PVN	
Zhang et al., 2012	MS	Rat	M	Juvenile	↑ AVP-mRNA in PVN and SON	
Veenema, 2009	MS	Rat	M	Juvenile	↑ AVP-mRNA in PVN	
Murgatroyd et al., 2009	MS	Mouse	M	Juvenile	↑ AVP-mRNA in PVN	mRNA expression
Murgatroyd et al., 2009	MS	Mouse	M	Adult	↑ AVP-mRNA in PVN	
Alcántara-Alonso et al., 2017	MS	Rat	M	Adult	↑ AVP-mRNA in PVN	
Veenema et al., 2006	MS	Rat	M	Adult	↑ AVP-mRNA in PVN and SON	
Rice et al., 2008	LBN	Mouse	M	Adult	No change in AVP-mRNA in PVN	

Wang et al., 2014	PD	Mandarin vole	M/F	Infant	↑ AVP-ir in PVN and SON	
Tsuda et al., 2011	MS	Mouse	M	Juvenile	↓ AVP-ir in PVN	
Veenema et al., 2007	MS	Mouse	M	Adult	↑ AVP-ir in PVN	
Veenema et al., 2007	MS	Mouse	F	Adult	No change in AVP-ir in PVN	
Wei et al., 2013	PI	Mandarin vole	M	Adult	↓ AVP-ir in PVN	
Desbonnet et al., 2008	MS	Rat	M	Adult	↓ AVP-ir in PVN	
Desbonnet et al., 2008	MS	Rat	F	Adult	No change in AVP-ir in PVN	Immunoreactivity
Veenema et al., 2006	MS	Rat	M	Adult	↑ AVP-ir in PVN and SON	
Zhang et al., 2012	MS	Mouse	M	Adult	↑ AVP-ir in PVN and SON	
Zhang et al., 2012	MS	Rat	M	Infant	↑ Fos-expression in AVP+ neurons in PVN, SON	
Hernández et al., 2016	MS	Rat	M	Adult	↑ AVP innervation density from PVN to amygdala	
Zhang et al., 2012	MS	Rat	M	Adult	↑ Volume of AVP-SON and AVP-PVN nuclei	

*For each reference (first column), the table indicates the ES model used, the species, gender (M, male; F, female) and the animal age-group, followed by the observed changes in the AVP system. Arrows indicated the direction of the effects (increase: up arrow; decrease: down arrow). If a reference is repeated, it is to mention different groups of animals in the same publication. M, male; F, female; MS, maternal separation; PVN, hypothalamic paraventricular nucleus; SON, hypothalamic supraoptic nucleus; LBN, limited bedding and nesting; PD, paternal deprivation; AVP-ir, arginine vasopressin immunoreactivity; PI, post-natal isolation; AVP+, arginine vasopressin positive.*

**TABLE 4:** OXT changes in the PVN and SON after ES.

**References**	**Model**	**Species**	**M/F**	**Age**	**OXT system changes**	
Wang et al., 2014	PD	Mandarin vole	M/F	Infant	↓ OXT-ir in PVN and SON	
Tsuda et al., 2011	MS	Mouse	M	Juvenile	↑ OXT-ir in PVN	
Wei et al., 2013	PI	Mandarin vole	M	Adult	↓ OXT-ir in PVN	Immunoreactivity
Veenema et al., 2007	MS	Mouse	M	Adult	No change in OXT-ir in PVN	
Veenema et al., 2007	MS	Mouse	F	Adult	↓ OXT-ir in PVN	

*Conventions as in Table 3. M, male; F, female; PD, paternal deprivation; OXT-ir, oxytocin immunoreactivity; PVN, hypothalamic paraventricular nucleus; SON, hypothalamic supraoptic nucleus; MS, maternal separation.*

In the PVN, ES consistently (18 out of 22 experimental groups) causes alterations in AVP (Table 3). ES most often (seven out of nine experimental groups tested) induces an increase in AVP-mRNA in the PVN (Veenema et al., 2006; Veenema and Neumann, 2009; Zhang et al., 2012; Murgatroyd et al., 2015; Alcántara-Alonso et al., 2017) (Table 3). These effects are found when AVP-mRNA is measured in infancy (Zhang et al., 2012), at juvenile age (Veenema and Neumann, 2009; Zhang et al., 2012; Murgatroyd et al., 2015) and in adulthood (Veenema et al., 2006; Murgatroyd et al., 2009; Alcántara-Alonso et al., 2017), making them consistent over development. Interestingly, only one study failed to find effects of ES on AVP-mRNA in the PVN, which was measured after use of limited bedding material instead of MS as a model of ES (group 05; 06 in Supplementary Table S1) (Rice et al., 2008). Similarly, in the SON, changes in AVP have been consistently reported (seven out of seven experimental groups) (Table 3). Here as well, increase of AVP-mRNA has been frequently observed (three out of three experimental groups) (Veenema et al., 2006; Zhang et al., 2012). Together, these initial results suggest that ES causes enhanced AVP synthesis in the PVN and SON, although increased AVP-mRNA expression does not necessarily translate to a respective increase in AVP protein expression in these regions.

This is underscored by the immunohistochemical results, which are less consistent than those at the mRNA level (Table 3). In the PVN, seven out of nine experimental groups show changes in AVP-ir after ES. AVP-ir in the PVN was increased in four out of nine experimental groups (Veenema et al., 2006; Veenema et al., 2007; Wang et al., 2012; Zhang et al., 2012), but decreased in three experimental groups (Desbonnet et al., 2008; Tsuda et al., 2011; Wei et al., 2013), without a clear link between directionality, species and age. The discrepancies between the mRNA and protein level may partially be explained by the qualitative rather than quantitative nature of immunohistochemistry. Furthermore, there are a variety of regulatory mRNA modifications governing transcript processing and translation, which affect the extent to which mRNA changes are translated into protein expression changes, and thus correspondence between results at the mRNA and protein level. Interestingly, these regulatory mechanisms themselves may be critically altered by stress, underscoring the complexity of the multi-level effects of ES (Engel et al., 2017). The remaining two experimental groups showed no changes in AVP-ir. Interestingly, these experimental groups were females only. The lack of effect in all female groups tested, suggests a possible effect of gender on AVP-ir (Veenema et al., 2007; Desbonnet et al., 2008). Several studies that measured AVP-ir in the PVN have also looked at AVP-ir in the SON (Table 3). Here, we see that three out of three experimental groups show changes in AVP-ir, all of them indicating increased AVP protein expression (Veenema et al., 2006; Wang et al., 2012; Zhang et al., 2012). Furthermore, ES is associated with enhanced activity of AVP-positive neurons in the PVN and SON of infant rats, and increased volume of AVP nuclei in the PVN and SON of adult rats (Zhang et al., 2012). Moreover, AVP innervation density from the PVN to the CeA is increased as a result of ES (Hernández et al., 2016).

Early life stress has also been shown to affect the OXT system in the PVN (seven out of nine experimental groups) and to a lesser extent in the SON (one out of one experimental group) (Table 4). Although studies at the level of OXT-mRNA are lacking, OXT-ir is more often measured as a consequence of ES, and in the PVN broadly shows decreased OXT expression (three out of five experimental groups) (Veenema et al., 2007; Wei et al., 2013; Wang et al., 2014). Only in one out of five experimental groups there was an increase in OXT-ir in the PVN (Tsuda et al., 2011), while in another there was no significant change (Veenema et al., 2007). Interestingly, this lack of effect was found in the male experimental group of this study, yet the female experimental group showed decreased OXT expression. Females may thus be more likely to react to ES with changes in OXT. OXT-ir in the SON has only been characterized in one study and had been decreased as consequence of ES (Wang et al., 2014).

Overall, the aforementioned studies suggest that ES induces changes in AVP in the PVN in terms of mRNA and protein expression levels, and in OXT at the level of protein expression. In the SON, there is more causal evidence for changes in AVP than for OXT. Taken together, the studies also suggest that AVP protein expression changes are more often found in males, and that most studies in the PVN and SON indicate a potentiation of AVP. With regards to OXT a seemingly different picture emerges. Although results are less consistent, changes in OXT in the PVN and SON do occur, and this seems to go in the other direction, i.e., decreased OXT expression. Moreover, contrary to male sensitivity to AVP changes, we suggest females may be more sensitive to changes in OXT after ES.

#### Causality of AVP and OXT Within the PVN and SON for Social Behavior

From inspection of Figure 1, we can see that the remainder of the two first rows is a lot less conclusive. That is, for neither the PVN nor SON, there is substantial evidence that AVP and OXT are causally related to aggression, social recognition, or social motivation. We will discuss the causal studies that are included in Figure 1 for the PVN and SON, and address additional literature that helps us understand the relationship between AVP and OXT in the PVN and SON, and our behaviors of interest.

##### Aggression

Although several studies have established a link between AVP and male offensive aggression (Blanchard et al., 2005; Ferris et al., 2006) and OXT and maternal aggression (Bosch, 2005), few studies have focused on the PVN and SON specifically. In terms of AVP signaling in male aggression, no causal studies exist for either the PVN or SON, but there are several correlational studies that implicate these regions as potential regions of interest in the future. In socially inexperienced mice, increased aggression is associated with enhanced AvpR1a expression in the PVN (Elliott Albers et al., 2006), and in models of pathological aggression, offensive aggression is associated with an enhanced Fos activation in AVP neurons of the SON (David et al., 2004; Albers, 2012). Cross-fostering of an aggressive mouse strain with a less aggressive strain results in a reduction of aggressive behavior as well as reduced AVP-ir in the SON (Bester-Meredith and Marler, 2001). Together, these correlational studies indicate that AVP signaling in the PVN and SON are an interesting target for the study of causal effects in the future.

There is more evidence on the role of OXT in maternal aggression, and these include experimental studies in the PVN. As can be seen from Figure 1, in three experimental groups, manipulation of OXT changes maternal aggression. In the PVN of some rat strains, OXT promotes maternal aggression, as blocking of OXTR in the PVN decreases maternal aggression in lactating dams (Bosch et al., 2004, 2007). In other strains, however, blocking paraventricular OXTR results in increased aggression, indicating that OXT is related to maternal aggression, but not in a unidirectional manner (Bosch et al., 2004; Bosch and Neumann, 2012). Finally, acute reduction of OXT synthesis in the PVN using antisense administration increases aggression in lactating dams (Giovenardi et al., 1998). Currently, no studies, causational or otherwise, have characterized the role of OXT in the SON in relation to maternal aggression, thus not allowing us to assess the potential effects of OXT changes on behavior. We can, however, conclude that in the PVN, alterations in OXT signaling are causally related to alterations in maternal aggression. Thus, previously discussed studies in females showing ES-induced reductions in paraventricular OXT may indeed translate to (bidirectional) alterations in maternal aggression.

##### Social recognition

There are also only very few studies directly assessing AVP and OXT in the PVN and SON in relation to social recognition. Electrical and osmotic stimulation of the PVN and SON in rats results in enhanced release of AVP in the hypothalamus, and this facilitates social recognition (Engelmann and Landgraf, 1994). Moreover, application of secretin to the SON activates OXT neurons and facilitates OXT release, which facilitates social recognition as well (Takayanagi et al., 2017). These studies point toward a role for AVP and OXT synthesis in social recognition, but used less specific ways of targeting AVP and OXT neurons. Thus it cannot be fully excluded that increased AVP and OXT were not mediated by another factor. More causational studies are required to study this link in more detail, and predict the effect of ES-mediated changes in the PVN and SON on social recognition.

##### Social motivation

No causational studies exist for AVP and OXT signaling in the PVN and SON and also correlational evidence on social motivation is sparse. With regards to the latter, high social interaction mice exhibit greater expression of PVN AVP and OXT mRNA compared to low interaction mice (Murakami et al., 2011), and time spent in social interaction correlates positively with the amount of AVP and OXT mRNA expression in the PVN (Flanagan et al., 1993; Murakami et al., 2011). It is currently unclear whether these expression differences are behaviorally causative, meriting caution in the interpretation of these effects, and the prediction of changes in social motivation after ES due to AVP/OXT changes in the PVN and SON.

#### Conclusion

We conclude that although there is abundant causational evidence that ES changes AVP and OXT systems in the PVN and SON, there is not enough evidence to reliably link these changes to potential behavioral outcomes in aggression, social recognition and social motivation. Only an effect of ES on maternal aggression via OXT changes can be experimentally supported. Correlational studies indicate that the PVN and SON may be relevant for other social behaviors through AVP and OXT signaling, and merit causal investigation.

### Medial Preoptic Nucleus/Anterior Hypothalamus

While for the PVN and SON, we have causal evidence for an effect of ES on AVP and OXT systems but little evidence for an effect of AVP or OXT on social behavior, the reverse is true for the MPOA/AH. Here, we have comparatively strong evidence for behavioral effects of AVP and OXT manipulations but we lack studies systematically exploring the effect of ES on AVP/OXT in this area. Although evidence is sparse and less consistent, Figure 1 and Supplementary Table S1 shows that several neighboring hypothalamic regions (the HAA, VMH, and LH) have also been studied in relation to ES and show encouraging changes in AVP and OXT systems. However, currently, the MPOA/AH is the only hypothalamic region that has been causally associated with our social behaviors of interest. We will therefore focus on the MPOA/AH, and argue that ES may affect OXT signaling in the MPOA/AH. Given the potential role for OXT signaling in the MPOA/AH in regulating aggression and social recognition, these behaviors may be affected as a consequence. However, the current state of the evidence merits caution in interpreting these links.

#### Causality of ES Effects on OXT in the MPOA/AH

As a consequence of ES, OXTR binding is increased in the MPOA/AH of adolescent male rats (Lukas et al., 2010). This change appears to be age-related, as it is not found in other age cohorts. Other evidence for changes in OXT system in MPOA/AH comes from a study in mandarin voles, in which ES causes a decrease in OXTR-mRNA in the MPOA/AH of males and females (Yu et al., 2015). Together, these results suggest that OXT signaling in the MPOA/AH may be susceptible to change as a result of ES, but that more causal studies are required to assess the extent and nature of this susceptibility.

#### Causality of OXT Within the MPOA/AH for Social Behavior

##### Aggression

Currently, one study manipulating OXT has suggested a causal role for OXT signaling in the MPOA/AH in regulating maternal aggression, reflected in Figure 1 (groups 045–055 in Supplementary Table S1). More specifically, site-specific injections of OXT into the MPOA/AH of female hamsters reduced maternal aggression in a dose dependent manner when tested immediately after, but not 30 min after injection. Conversely, when injected into the MPOA/AH with an OXTR antagonist, maternal aggression is increased when tested 30 min, but not immediately after injection (Harmon et al., 2002). Although currently the only causational study, it provides initial evidence that OXT signaling in the MPOA/AH is important in regulating maternal aggression.

##### Social recognition

Figure 1 shows that the MPOA/AH has been assessed in one study to establish the causal role of OXT in social recognition (groups 056–061 of Supplementary Table S1). Injection of OXT into the MPOA/AH has, at a variety of dosages, a facilitating effect on social recognition (Popik and van Ree, 1991). A role for OXT signaling in the MPOA/AH in social recognition is further supported by a correlational study: in high- versus low- recognition mice, the MPOA/AH is one of the regions where differential OXT expression can be observed (lower in low-recognition mice) (Clipperton-Allen et al., 2012).

##### Social motivation

We are currently unaware of any studies that have specifically assessed the association between OXT in the MPOA/AH and social motivation in neither causal nor correlational studies. If OXT signaling in the MPOA/AH modulates rewarding aspects of interactions, is not clear and needs further investigation.

#### Conclusion

Although not consistently found, some studies report OXT system changes in the MPOA/AH after ES. Initial evidence indicates that OXT signaling in the MPOA/AH is involved in regulation of maternal aggression (OXT reduces maternal aggression) and social recognition (OXT enhances social recognition). However, it is difficult to predict whether the OXT system changes after ES might relate to behavioral dysfunction, as on the one hand OXTR binding in the MPOA/AH is increased, and on the other hand, OXTR-mRNA in the MPOA/AH is decreased as a consequence of ES. Thus, we can generally conclude that the MPOA/AH OXT system may be susceptible to (bidirectional) changes after ES, which may affect maternal aggression and social recognition.

### Lateral Septum

Figure 1 shows that some evidence indicates ES causes changes in AVP and OXT in the LS. Moreover, given the potential role of AVP and OXT in social recognition and social motivation (Figure 1), we see preliminary indications that ES-induced changes in the LS could be causally linked to altered social behavior, primarily in context of AVP changes and social recognition and social motivation. As there is lack of evidence (OXT) and counterevidence (AVP) for the link between septal AVP and OXT signaling regulating aggression (see Figure 1 and Supplementary Table S1), we will not discuss this behavior further, as no changes are expected.

#### Causality of ES Effects on AVP and OXT in the LS

Several changes in AVP and OXT occur after ES in the LS, but these are not all unidirectional. Although there is an increase in AvpR1a binding in juvenile male rats, this increased binding is no longer reported in later age cohorts (Lukas et al., 2010). In adult male rats, there is a decrease in AVP release in the LS (Lukas et al., 2011a). This study has been mentioned before as it has linked this reduction in septal AVP to impairment in social recognition after ES. Moreover, retro-dialysis of AVP into the septum rescued the impairment. Although not always reported (Barrett et al., 2015), OXTR also shows some changes in the LS, as binding is reduced after ES, which was associated with impairment in social recognition (Cao et al., 2014). Together, these studies provide strong indication that changes in primarily AVP, and to a lesser extent OXT signaling in the LS, after ES are related to deficits in social recognition. The evidence offered below further supports this notion.

#### Causality of AVP and OXT Within the LS for Social Behavior

##### Social recognition

Arginine vasopressin signaling in the LS has been repeatedly shown to be crucial for social recognition: site-specific injections of AVP to the LS, as well as septal AVP administration using retro-dialysis, consistently facilitates social recognition (Engelmann and Landgraf, 1994; Landgraf et al., 2003; Veenema et al., 2012), whereas administration of an AvpR1a antagonist into the LS blocks the formation of social recognition memory (Engelmann and Landgraf, 1994; Landgraf et al., 1995, 2003; Everts and Koolhaas, 1997, 1999; Bielsky et al., 2005; Veenema et al., 2012). Moreover, enhancement/overexpression of AvpR1a in the LS facilitates social recognition (Landgraf et al., 2003; Bielsky et al., 2005), and reintroduction of AvpR1a into the LS of AvpR1a knockouts completely rescues the previously impaired behavior (Bielsky et al., 2005). Only in juvenile animals no effect of AVP manipulation in the LS has been reported (Veenema et al., 2012).

For OXT signaling in the LS, the causal involvement in social recognition is less established. OXT injection into the LS has been reported to cause no change in social recognition at a different dosages (Popik and van Ree, 1991), and OXTR antagonism in the LS has been observed to lead both to abolished social recognition (Lukas et al., 2013), as well as no change (Landgraf et al., 2003). Thus, whether or not OXT signaling in the LS is causal in social recognition is currently not fully supported.

##### Social motivation

AvpR1a activation in the LS is, in a consistent manner, associated with social motivation. That is, overexpression of AvpR1a in the LS causes an increase in active social interactions (Landgraf et al., 2003), whereas site-specific injections of AvpR1a antagonists in the LS decrease social interactions (Beiderbeck et al., 2007; Veenema et al., 2012). An exception is the study by Leroy et al. (2018) reporting that an AvpR1b antagonist vastly reduced social aggression and thereby increased the fraction of mice displaying only social investigation. The role of AvpR1a in regulating social motivation has also been supported by correlational studies. Male prairie voles that interact with conspecifics at a high level exhibit a relative increase in AvpR1a in the LS relative to counterparts displaying less social motivation (Ophir et al., 2009).

#### Conclusion

There are indications that the LS is sensitive to changes in both AVP and OXT systems after ES, pointing slightly to a depression of both in adults. Given the established facilitating role of AVP signaling in social recognition and social motivation, a decrease in septal AVP is expected to affect both behaviors negatively. Conversely, the link between OXT and social recognition is less well established, and although decreased binding as well as social recognition deficits have been found simultaneously after ES, whether these effects are causal is unsure.

### Amygdala

From inspection of Figure 1, we see evidence that no changes in the CeA occur after ES, and that there is limited evidence for changes in the MeA, which has been reported only in OXT signaling. As OXT in the MeA does not seem related to aggression and social motivation (see Figure 1 and Supplementary Table S1), we will only discuss the evidence for oxytocinergic signaling in the MeA regulating social recognition.

#### Causality of ES Effects on OXT in the MeA

Although no changes are reported in OXTR expression at three different ages in the MeA (Lukas et al., 2010), in male and female adult mandarin voles, OXTR-mRNA is decreased after ES (Cao et al., 2014). This is associated with a decrease in social recognition in this study. From Figure 1, and as we argue below, these changes in OXTR are likely instrumental in the behavioral deficit, given the causal role of OXT in the MeA for social recognition.

#### Causality of OXT Within the MeA for Social Behavior

##### Social recognition

Modulation of OXT at the level of the MeA seems critical for social recognition (Stoop, 2012). Although infusion of OXT facilitates social recognition in several regions, only in the MeA it is thought to be necessary and sufficient for social discrimination (Gabor et al., 2012). In OXT-knockout mice with impaired social recognition, OXT infusions into the MeA rescues this behavior (Ferguson et al., 2002). Moreover, an OXT antagonist injected into the MeA blocks normal social recognition memory in WT male mice (Ferguson et al., 2001). Also in females, blocking OXTR in the MeA through antisense oligonucleotides impairs normal social recognition (Choleris et al., 2007). Furthermore, facilitating effects of secretin on social recognition are blocked by applying an OXTR antagonist to the MeA (Takayanagi et al., 2017) (not included in Figure 1). Together, these findings suggest that the MeA is crucial for OXT mediated social recognition.

#### Conclusion

The above evidence suggests that ES may result in reduced oxytocinergic signaling in the MeA, and that if these changes occur, they are likely causative in affected social recognition, since OXT signaling in the MeA is necessary and sufficient for this behavior.

### Other Basal Forebrain Regions: Bed Nucleus of the Stria Terminalis (BNST) and Nucleus Accumbens (NAcc)

Figure 1 indicates that in the BNST and NAcc, changes in AVP and OXT, respectively, occur after ES, but are not consistently found. Moreover, AVP and OXT signaling within these regions are only weakly linked to a limited amount of behaviors. We will therefore discuss both only shortly below.

#### Causality of ES Effects on AVP and OXT in the BNST and NAcc, and Association to Social Behavior

Although in the BNST AVP-mRNA is increased after ES (Veenema and Neumann, 2009), in three other cohorts of different ages, no changes in AvpR1a have been found (Lukas et al., 2010). Changes in AVP signaling in the BNST, although not consistently found, can only be weakly linked to potential changes in aggression and social motivation. That is, administration of AVP in the BNST enhances aggression in one experimental group, whereas blocking AvpR1a in the BNST (Veenema et al., 2010), and lesioning BNST AVP neurons (Rigney et al., 2019) did not affect aggression in other studies. In females, however, blocking AvpR1a in the BNST reduced maternal aggression (Bosch et al., 2010). Changes in AVP are more consistently linked to social motivation. Lesioning BNST AVP neurons reduced social interaction in males, but not females in one study (Rigney et al., 2019), whereas an AvpR1a antagonist injected into the BNST reduced social motivation in both sexes in another (Duque-Wilckens et al., 2016). No changes in OXT in the BNST have been found after ES.

Conversely, in the NAcc, there is no evidence for changes in AVP, and mixed evidence for altered OXT signaling after ES, as indicated by a decrease in OXTR expression in one experimental group (Cao et al., 2014), and no change in OXTR binding in another (Barrett et al., 2015). If indeed ES changes OXT signaling in the NAcc, this may have consequences for social motivation. In Figure 1, the experimental groups reflect a dose-response relationship between OXT administration to the NAcc and social motivation (groups 093–095 of Supplementary Table S1), with increased social approach behavior at medium dosage (Yu et al., 2016). An OXTR antagonist administered to the NAcc on the other hand reduces social approach, at higher, but not lower dosages (Yu et al., 2016) (groups 096–098 of Supplementary Table S1). Although using a slightly different paradigm than the one most frequently used in other studies and described in Box 2, a similar association between OXT in the NAcc and social motivation has been studied by using conditioned place preference with a social stimulus animal. Here, OXTR antagonist infusion into the NAcc reduced social conditioned place preference (Dölen et al., 2013).

#### Conclusion

Evidence on the BNST and NAcc is rather inconclusive, as changes after ES and changes in behavior are not frequently or consistently reported. Particularly in the BNST, there is more evidence for lack of change than for change in AVP and OXT systems after ES. If changes would occur, it may affect social motivation, and to a lesser extent, aggression. In context of the NAcc, the state of the evidence is mixed, and if changes in OXT systems occur, this might relate to potential alterations in social motivation, given the role of OXT signaling in regulating this behavior. As a reduction in OXT signaling has been found as a consequence of ES, this may cause reduced social motivation.

### Causal Pathways Linking ES to Social Behavior: Conclusion

**FIGURE 2:**
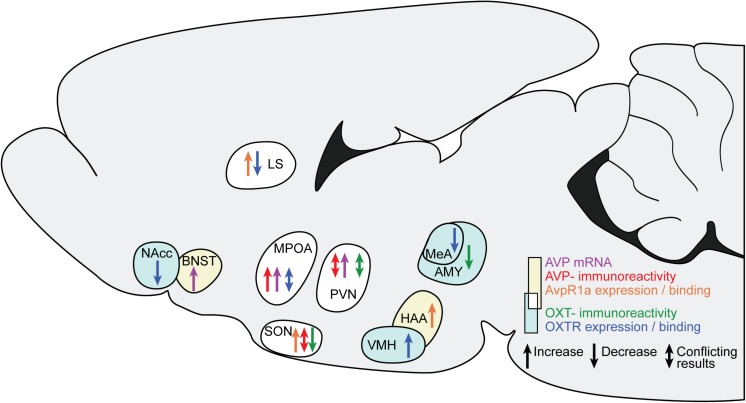
Changes in AVP and OXT in brain regions after ES. The sagittal slide schematically illustrates the brain regions showing AVP and OXT changes after ES. Yellow background and warm colors in the arrows indicates regions and effects associated with AVP, light cyan and cold colors indicates regions associated with OXT. The white background indicates regions associated with both AVP and OXT. LS, lateral septum; BNST, bed nucleus of the stria terminals; MPOA, medial preoptic nucleus; SON, hypothalamic supraoptic nucleus; PVN, hypothalamic paraventricular nucleus; VMH, ventromedial hypothalamus; HAA, hypothalamic attack area; MeA, medial amygdala.

Figure 1 and our discussion of the available causal data indicate that several changes in AVP and OXT occur after ES, across diffuse brain regions. All studies finding an effect of ES on AVP and OXT are summarized in Figure 2.

Figure 1 and the discussion of its exemplar data also indicate that the study of brain areas that mediate ES effects is unevenly spread across the different regions. Although there is a lot of evidence for changes after ES in the PVN and SON, these regions are not examined extensively in context of aggression, social recognition, and social motivation. In many extrahypothalamic regions, the number of experimental groups is far less in context of ES research on AVP and OXT system changes, although for some of these, particularly the MPOA/AH, LS and MeA, a link to one or more of the social behaviors is relatively well established. Combined with the evidence that in some, but not all groups, AVP and OXT systems are altered as a consequence of ES within these regions, these studies provide initial indications for mediating effects of AVP and OXT. However, Figure 1 clearly signals the need for an expansion on the current framework through targeted studies on these regions after ES, as well as intervention studies to indicate causality. We furthermore propose that more research is needed on the mechanisms through which ES changes AVP and OXT systems, and the translational value of the current rodent work in humans. Initial research on both of these themes is outlined below.

## Mechanisms Through Which Early Postnatal Stress Alter AVP and OXT Systems

The previous section suggests that the AVP- and OXT-system display a high level of plasticity as a function of the early postnatal period. Likely, one of the mechanisms that convey cues from early environmental conditions to AVP and OXT physiology is through epigenetic changes. These mechanisms alter the likelihood of a gene being expressed through chemical modifications to chromatin, including DNA and histone protein modifications (Marsit, 2015). Especially DNA methylation is conceived as a persistent mediator of the accessibility of DNA, and thus an important determinant of the transcription rate of genes. Initial findings indicate that both the AVP and OXT systems are subject to epigenetic alterations as a consequence of ES, although few studies have used experimental manipulations in rodents or other animal model species. In mice, ES has been associated with a reduction in methylation at the CGI3 region, a regulatory region of the AVP gene (Murgatroyd et al., 2009). This hypomethylation was found to last well into adulthood and was associated with increased AVP-mRNA in the PVN, which is in line with the frequent reports of enhanced AVP-mRNA in the PVN of rodents in other ES studies.

To date, rodent studies assessing epigenetic modifications in OXT(R)-genes after ES are scarce. We therefore reviewed evidence in other model species, as it has been assessed in rhesus macaques. These findings can be corroborated with correlational evidence on epigenetic changes in humans. In hippocampal samples of rhesus macaques, K3K4me3-binding levels at the OXTR gene were significantly decreased in animals reared without parents, with a corresponding decrease in OXTR-mRNA expression (Baker et al., 2017). The latter results are in line with predominantly decreased OXTR-binding and expression in rodents as a result of ES.

Interestingly, in humans, associations have been reported between enhanced OXTR-methylation and exposure to ES in females, but not in males (Gouin et al., 2017). Moreover, in females, self-reported low maternal care was also associated with enhanced OXTR-methylation in the OXTR gene (Unternaehrer et al., 2015). Finally, abuse interacts with CpG methylation of OXTR genes to predict adult psychopathology (Smearman et al., 2016). It is important to note that these studies do not allow for causational inferences, highlighting the importance for additional studies manipulating the early life environment of animal models. However, together these lines of evidence indicate that ES, via epigenetic changes, has the potential to alter critical characteristics of the AVP- and OXT-systems.

## Translational Relevance: ES, AVP, OXT, and Social Behavior in Humans

Altogether the above-presented literature indicates that behavioral changes in aggression, social recognition and, to a lesser extent, social motivation, occur after ES, and that this is often accompanied by changes in OXT and AVP. Although this body of work is far from complete, particularly with regards to causality of the associated changes, it begs the question of whether we have evidence that the human OXT and AVP systems may mirror the sensitivity to ES found in rodents. Below, we briefly review the link between ES, OXT and AVP in humans. Preliminary evidence suggests that there is a link between ES and AVP and OXT changes in humans as well, but these often carry methodological limitations. Most studies rely on measures of peripheral AVP and OXT, and the relationship between these levels and central AVP and OXT is heavily debated (Meyer-Lindenberg et al., 2011). Although the validity can be challenged, reduced physical and emotional contact early in life is associated with lowered peripheral plasma AVP levels in children later on (Fries et al., 2005), and lower CSF OXT concentrations have been found in adult women with a history of childhood abuse (Heim et al., 2009).

Some studies have also revealed epigenetic alterations as a consequence of ES in OXTR expression; in females, enhanced OXTR methylation is associated with exposure to ES (Gouin et al., 2017), and self-reported low maternal care (Unternaehrer et al., 2015). Moreover, early abuse interacts with CpG methylation of OXTR genes to predict adult psychopathology (Smearman et al., 2016). Thus, there are preliminary indications that ES co-varies with changes in proxies of the AVP and OXT system in humans, and that these changes may have a role in predisposing to psychopathology.

So far, the nature of changes is obscure, as is the effect of these changes on social behavior. However, perturbed functioning of AVP and OXT has recently been linked to a variety of mental disorders with social dysfunction, and they are suggested to have therapeutic relevance for treatment of disorders with social dysfunction as well (Heinrichs et al., 2009; Meyer-Lindenberg et al., 2011). Interestingly, in healthy individuals, administration of OXT facilitates social behaviors classically impaired by ES in rodents. For example, post-learning administration of OXT enhances immediate and delayed recall of recognition of faces (Savaskan et al., 2008), and OXT specifically improves recognition memory for faces but not for non-social stimuli (Rimmele et al., 2009).

Trust, a perquisite for social approach and motivation in humans, is also facilitated by OXT administration: OXT specifically increases an individual’s willingness to accept social risks within social interactions, but not in non-social situations (Kosfeld et al., 2005). This has been replicated in two studies (Mikolajczak et al., 2010a,b). Additionally, intranasal OXT administration alters responses to the pain of others in the insula, a neural correlate of empathy (Bos et al., 2015) that has been associated with the inhibition of aggression and antisocial behavior (Hein et al., 2010; Meffert et al., 2013).

Less research has been conducted on modulation of AVP in relation to social behavior in humans. Two recent studies have examined the effects of intranasal AVP on human facial responses in males. Administration of AVP enhanced corrugator supercilii electromyogram responses to emotionally neutral faces in a way that was similar to placebo subjects’ magnitude of responses to angry facial expressions. Because this muscle group is crucial to species-specific agonistic social communication, it has been suggested that AVP modulates agonistic behavior in humans (Thompson et al., 2004). In a follow up study, AVP enhanced agonistic facial motor patterns and decreased subjective ratings of friendliness toward male faces, but only in male subjects (Thompson et al., 2006). Together, these results suggest that in humans, AVP influences social communication processes, but in a sex-specific manner.

Modulation of the OXT, and perhaps also of the AVP system, thus seems to have the potential to enhance behaviors that are typically affected in rodents as a consequence of ES. Moreover, OXT is being tested as a therapeutic tool to alleviate social dysfunction in mental disorders for which ES is a risk factor (Heinrichs et al., 2009; Meyer-Lindenberg et al., 2011). This research line is in its infancy, and has rendered some positive results on aspects of social functioning in autism, schizophrenia, and mood disorders (Hollander et al., 2007; Pincus et al., 2010; Pedersen et al., 2011; Yatawara et al., 2016; Parker et al., 2017). Perhaps most interesting in the context of ES is borderline personality disorder. Since borderline personality disorder is closely linked to childhood trauma and neglect, it has been suggested that it can be understood as a disorder of attachment, where ES interferes with the developing neuropeptide systems and alters the receptor binding of AVP and OXT, thereby promoting disorders of attachment like borderline personality disorder (Carter, 2003; Heinrichs et al., 2009).

Surprisingly, very little research has been done to assess potential therapeutic effects of OXT in borderline personality disorder. A recent study showed that OXT attenuates the stress response in borderline patients after a social stressor (Simeon et al., 2011). However, a different study indicated that OXT deceases trust in a small sample of borderline patients (Bartz et al., 2011). Currently, several clinical trials are in progress to assess the therapeutic potential of OXT in borderline personality disorder (see ClinicalTrials website^1^). It may be important to consider aspects of attachment or early life in such clinical trials, as they may mediate the therapeutic effects of OXT. For example, OXT enhances positive recollection of maternal care in securely attached individuals, whereas in anxiously attached individuals, OXT has the opposite effect (Bartz et al., 2010). Moreover, in males that have suffered early parental separation, stress responses are differentially attenuated after OXT treatment (Meinlschmidt and Heim, 2007), underscoring the importance of considering the early environment when assessing manipulation of these neuropeptides as a treatment option.

In conclusion, it remains largely unknown whether and how ES affects OXT and AVP in humans, whether this impacts brain areas related to social behaviors, and whether and how manipulations of OXT and AVP may be used to alleviate social symptoms. We advocate for aligned animal experimental and human studies to understand the neurobiological underpinnings of how adverse early life experiences impact later social behaviors.

## Discussion

In the present review, we have given an overview of the current state of causal evidence indicating that the effects of ES on social behavior later in life are – at least in part – mediated by AVP and/or OXT. Although several studies measured both AVP and OXT and social behavior after manipulation of the early environment, these studies are showing correlations rather than causality. We have therefore constructed a causality matrix in order to assess where in the brain, if at all, (1) ES alters AVP and/or OXT, and (2) changes in AVP and/or OXT causes changes in social behavior. We feel that at present, the evidence we have to support such causal pathways remains rather patchy. Although it indicates a susceptibility to the effects of ES on AVP and/or OXT in some regions, these cannot always be linked directly to altered behavior because we lack studies manipulating AVP and OXT systems in these regions while reading out the impact on social behavior.

Conversely, in other regions, the susceptibility to ES effects is not sufficiently studied. Thus, although we see indications of relevance of ES changes in AVP and OXT in regions like the PVN/SON, LS, and MPOA, we advocate for more studies to clarify the nature, extent, and causal role of changes in these regions, particularly by adding rescue experiments. Moreover, we advocate for more aligned rodent and human studies, propose that, to increase our understanding of the environmental contributions to variability and dysfunction in social behavior in context of AVP and OXT, some outstanding questions require answers (Box 3).

**BOX 3. Outstanding questions in research on the relation between ES, AVP/OXT, and social behavior.**

1. What are the effects of ES on epigenetic mechanisms and post-transcriptional modifications?
  Early life stress-induced changes in the epigenome could be a mechanism through which ES affects Avp(R1a) and OXT(R). Moreover, although acute stress is associated with alterations in post-transcriptional mechanisms, effects of chronic ES on Avp(R1a)- or OXT(R) genes specifically remain to be explored.
2. What are the effects of AVP and OXT on cellular and synaptic physiology in healthy and ES- conditions?
  There is currently little knowledge on how AVP and OXT modulate cellular excitability and synaptic characteristics, such as the synaptic strength between neurons in brain areas relevant for social behavior. Exploring how AVP and OXT exert their function on behavior in a model integrating molecular, cellular, and synaptic parameters would provide important information toward a truly mechanistic understanding of how ES alters social behavior.
3. How does ES affect the network dynamics in social brain circuits?
  So far, most studies have focused on single region changes in AVP and OXT after ES. However, aggression, social recognition and social motivation, are outputs resulting from coordinated activity in many different regions, many of which are modulated by AVP and/or OXT. How ES affects connectivity or synchronization between multiple hubs in social networks is unclear. Several intricate imaging methods now exist for small rodent species, and may provide a starting point to study network dynamics.
4. What is the relationship between peripheral and central levels of AVP and OXT?
  A variety of methodological concerns hamper assessing the relationship between ES, AVP/OXT and social behavior in humans, and potential interference with ES-induced changes. Human research largely relies on measurements of plasma levels of AVP and OXT. It is heavily debated whether these reflect central AVP and OXT. Moreover, it has been argued that OXT might be utilized to alleviate social dysfunction (after ES or otherwise). However, the extent to which intranasal OXT crosses the blood–brain barrier, and where it binds to neuronal receptors, is debated. These critical questions need to be answered before we can answer whether ES is associated with changes in AVP and OXT in humans, and whether interference with these changes may have treatment potential.

Our review has a number of limitations. First of all, we looked at changes in AVP and OXT in our causality matrix (effect vs. no effect), rather than the direction and manifestation of such changes. This was because studies investigate different indexes of OXT and AVP systems (mRNA, protein, immunoreactivity, etc.), and it is premature to attempt a quantitative meta-analysis given that most cells in our matrix would not contain multiple studies looking at the exact same index. This, however, limits the validity of our approach, as studies finding impacts of ES in opposite directions in different groups could indicate a true effect that is complicates by other variables (e.g., gender and age), or the presence of false positive fluctuations that one would expect to randomly distribute in both directions. Our analysis further ignores interactions between brain regions or with other hormonal and neurotransmitter systems. For example, AVP and OXT show interactions with testosterone, serotonin, and dopamine, and these interactions are also relevant for social behavior (Koolhaas et al., 1990; Ferris and Delville, 1994; Baskerville and Douglas, 2010). Finally, the restriction in the number of studies has prevented us from systematically assessing the differences between different models of stress induction (for example, classical maternal deprivation versus limited bedding and nesting), or the effect of sex and rodent species. We expect each of these factors to be relevant for the effects of ES on AVP and/or OXT, and on social behavior.

With these limitations in mind, we consider this review as carrying two main messages. First, there is encouraging evidence for a link between ES, AVP/OXT, and social behavior in rodents, which may provide inspirations for further validation and possible development of pharmacological therapies to rescue individuals from the terrible long-term impact of ES. Second, our analyses identify just how many cells of our causal matrix need further investigation and we hope that this may direct future efforts into promising directions.

## Author Contributions

NK, VG, CK, HK, and PL contributed to the conception and design of the manuscript. NK wrote the draft of the manuscript with revisions by HK, VG, CK, and PL. VG and NK provided the figures. All authors contributed to revision of the final manuscript and approved it for submission.

## Conflict of Interest Statement

The authors declare that the research was conducted in the absence of any commercial or financial relationships that could be construed as a potential conflict of interest.

## Footnotes

1 www.clinicaltrials.gov

## Abbreviations

AH: anterior hypothalamus
AVP: arginine vasopressin
AVP-ir: arginine vasopressin-immunoreactivity
AvpR1a: arginine vasopressin receptor 1a
AvpR1b: arginine vasopressin receptor 1b
BNST: bed nucleus of the stria terminalis
CeA: central amygdala
ES: early life stress
HAA: hypothalamic attack area
LH: lateral hypothalamus
LS: lateral septum
MeA: medial amygdala
MPOA: medial preoptic nucleus
MS: maternal separation
NAcc: nucleus accumbens
OXT: oxytocin
OXTR: oxytocin receptor
PD: paternal deprivation
PI: post-natal isolation
PVN: hypothalamic paraventricular nucleus
SON: hypothalamic supraoptic nucleus
VMH: ventromedial hypothalamus.
