# Supplementary material for: Early Life Adversity and Adult Social Behavior: Focus on Arginine Vasopressin and Oxytocin as Potential Mediators
Source: Front Behav Neurosci. 2019 Jul 26;13:143. doi: 10.3389/fnbeh.2019.00143 (PMC6676334; doi:10.3389/fnbeh.2019.00143)
Supplement: Supplementary file 1 [file Table_1.DOCX]

|  |  | | **Causal effects of AVP and OXT in social behavior** | | | | | | | | |
| --- | --- | --- | --- | --- | --- | --- | --- | --- | --- | --- | --- |
|  | **Causal effects of ES** | | **Aggression** | | | **Social recognition** | | | **Social motivation** | | |
| **Region** | AVP | OXT | | AVP | OXT | | AVP | OXT | | AVP | OXT |
| PVN | 001, 002, 003, 004, 005, 006, 007, 008, 009, 010, 011, 012, 013, 016, 017, 018, 019, 020, 021, 022, 023, 029 | 013, 016, 017, 018, 019, 020, 021 | |  | 033, 034, 035, 157 | | 036, 037^1^ |  | |  |  |
| SON | 001, 002, 003, 004, 011, 012, 013 | 013 | |  |  | | 036, 037^1^ |  | |  |  |
| MPOA/AH |  | 024, 025, 026^2^ 031 | | 038, 039, 040, 041, 042, 043, 044, 152 | 045, 046, 047, 048, 049, 050, 051, 052, 053, 054, 055^3^ | | 062, 063, 064, 065^4^ | 056, 057, 058, 059, 060, 061^4^ | |  |  |
| LH | 019, 020, 024, 025, 026^2^ | 019, 020 | |  |  | |  |  | |  |  |
| VMH |  | 024, 025, 026^2^ | |  |  | |  |  | |  |  |
| HAA | 024, 025, 026^2^ |  | |  |  | |  |  | |  |  |
| CeA | 024, 025, 026^2^ | 024, 025, 026^2^ 032 | | 069, 070 | 071, 073, 074, 076, 077, 078, 079, 080, 158 | |  |  | |  | 073, 074, 081, 082, 083, 084^5^ 089, 090, 155, 156 |
| MeA |  | 024, 025, 026^2^ 030 | | 099, 100, 101, | 075 | | 124, 159, 160 | 102, 103, 104, 105, 154 | |  | 085, 086, 087, 088^5^  091, 092 |
| LS | 024, 025, 026^2^ 028, | 024, 025, 026^2^ 032 | | 108, 109, 112, 113, 114, 115, 161 |  | | 116, 117, 118, 119, 120, 123, 125, 126, 127, 128, 129, 131, 132, 133, 143, 144, 145, 146, 147, 148 | 066, 067^4^ 106, 107, 121 | | 115, 122, 147, 148, 162 |  |
| BNST | 007, 024, 025^2^ 026 | 024, 025, 026^2^ 032 | | 110, 111, 134, 153, 163 | 072 | |  | 135, 136, 137, 138 | | 164, 165, 166, 167 | 139, 140, 141, 142, 149, 150 |
| NAcc |  | 030, 032 | |  |  | |  |  | | 168 | 093, 094, 095, 096, 097, 098^6^  151 |

**Table S1. Causality table for effects of ES on AVP and OXT, and AVP and OXT manipulation on social behavior.** For each region of interested listed in the first column, the table reports in green the experimental groups supporting a causal effect of ES manipulation on AVP and OXT (second and third columns) or an effect of an AVP or OXT manipulation on the three behaviors of interests (last 6 columns), and in red the experimental group that bring no significant evidence for a causal effect. See Figure 1 for a graphic illustration of this table, and Table 1 for abbreviations.

Footnotes: ^1^036 and 037: experimental groups in which PVN and SON were stimulated, which lead to an increase in AVP in the LS, and change in social recognition. ^2^024-026: age cohorts from the same study at juvenile (024), adolescent (025) and adult (026) age. ^3^045-055: experimental groups from the same study, reflecting different dosages of OXT at different times (045-050) and of OXTR at different times (051-055). ^4^056-065: experimental groups from the same study reflecting OXT or OXTR antagonist treatment to the MPOA (056-061) or LS (066-077) at different dosages, or AVP treatment to the MPOA at different dosages (062-065). ^5^081-088: experimental groups from the same study reflecting OXT or OXTR treatment to the CeA (081-084) or MeA (085-088) in males and females. ^6^093-098: experimental groups from the same study reflecting OXT or OXTR antagonist treatment at different dosages.

**Table S2. References to experimental groups in Figure 1 and causality Table S1.** For each experimental group (third column) included in Figure 1 and causality table S1, the table reports first author and year of publication (first column) and title of paper (second column) from which the experimental group was derived. Experimental group numbers correspond to group numbers as reported in Table S1. An experimental group is defined as an experimental unit within a specific study.

| **First author, year** | **Title** | **Experimental group(s)** |
| --- | --- | --- |
| Zhang, 2012 | Hypothalamic vasopressin system regulation by maternal separation: Its impact on anxiety in rats | 001, 002, 003, 004 |
| Rice, 2008 | A novel mouse model for acute and long-lasting consequences of early life stress | 005, 006 |
| Veenema, 2009 | Maternal separation enhances offensive play-fighting, basal corticosterone and hypothalamic vasopressin mRNA expression in juvenile male rats | 007 |
| Murgatroyd, 2009 | Dynamic DNA methylation programs persistent adverse effects of early-life stress | 008, 009 |
| Alcantara-Alonso, 2017 | Altered functionality of the corticotrophin-releasing hormone receptor-2 in the hypothalamic paraventricular nucleus of hyperphagic maternally separated rats | 010 |
| Veenema, 2006 | Effects of early life stress on adult male aggression and hypothalamic vasopressin and serotonin | 011, 012 |
| Wang, 2012 | Early-life stress-induced anxiety-related behavior in adult mice partially requires forebrain corticotropin-releasing hormone receptor 1 | 013 |
| Oreland, 2010 | Short- and long-term consequences of different early environmental conditions on central immunoreactive oxytocin and arginine vasopressin levels in male rats | 014, 015 |
| Tsuda, 2011 | Early life stress disrupts peripubertal development of aggression in male mice | 016, 017, 018 |
| Veenema, 2007 | Opposite effects of maternal separation on intermale and maternal aggression in C57BL/6 mice: Link to hypothalamic vasopressin and oxytocin immunoreactivity | 019, 020 |
| Wei, 2003 | Neonatal tactile stimulation alleviates the negative effects of neonatal isolation on novel object recognition, sociability and neuroendocrine levels in male adult mandarin voles (Microtus mandarinus) | 021 |
| Desbonnet, 2008 | Sexually dimorphic effects of maternal separation stress on corticotrophin-releasing factor and vasopressin systems in the adult rat brain | 022, 023 |
| Lukas, 2009 | Maternal separation interferes with developmental changes in brain vasopressin and oxytocin receptor binding in male rats | 024, 025, 026 |
| Lesse, 2017 | Chronic Postnatal Stress Induces Depressive-like Behavior in Male Mice and Programs second-Hit Stress-Induced Gene Expression Patterns of OxtR and AvpR1a in Adulthood | 027 |
| Lukas, 2011 | Early life stress impairs social recognition due to a blunted response of vasopressin release within the septum of adult male rats | 028 |
| Hernandez, 2016 | Hypothalamic Vasopressinergic Projections Innervate Central Amygdala GABAergic Neurons: Implications for Anxiety and Stress Coping | 029 |
| Cao, 2014 | Neonatal paternal deprivation impairs social recognition and alters levels of oxytocin and estrogen receptor α mRNA expression in the MeA and NAcc, and serum oxytocin in mandarin voles | 030 |
| Yu, 2015 | Early bi-parental separation or neonatal paternal deprivation in mandarin voles reduces adult offspring paternal behavior and alters serum corticosterone levels and neurochemistry | 031 |
| Barret, 2015 | The oxytocin system promotes resilience to the effects of neonatal isolation on adult social attachment in female prairie voles | 032 |
| Bosch, 2005 | Brain Oxytocin Correlates with Maternal Aggression: Link to Anxiety | 033, 034, 079, 080, 161 |
| Giovenardi, 1998 | Hypothalamic paraventricular nucleus modulates maternal aggression in rats: Effects of ibotenic acid lesion and oxytocin antisense | 035 |
| Engelmann, 1994 | Simultaneous monitoring of intracerebral release and behavior: vasopressin improves social recognition | 036, 037 |
| Ferris, 1988 | Vasopressin receptor blockade in the anterior hypothalamus suppresses aggression in hamsters | 038, 039, 040 |
| Portegal, 1989 | Intraspecific aggression in male hamsters is inhibited by intrahypothalamic vasopressin‐receptor antagonist | 043, 044 |
| Harmon, 2002 | Oxytocin inhibits aggression in female Syrian hamsters | 045, 046, 047, 048, 049, 050, 051, 052, 053, 054, 055 |
| Popik, 1991 | Oxytocin but not vasopressin facilities social recognition following injection into the medial preoptic area of the rat brain | 056, 057, 058, 059, 060, 061, 062, 063, 064, 065, 066, 067 |
| Bosch, 2010 | Vasopressin released within the central amygdala promotes maternal aggression | 069, 070 |
| Consiglio, 2005 | Effects of oxytocin microinjected into the central amygdaloid nucleus and bed nucleus of stria terminalis on maternal aggressive behavior in rats | 071, 072 |
| Calcagnioli, 2015 | Oxytocin microinjected into the central amygdaloid nuclei exerts anti-aggressive effects in male rats | 073, 074 |
| Lubin, 2003 | An oxytocin antagonist infused into the central nucleus of the amygdala increases maternal aggressive behavior. | 076 |
| Ferris, 1992 | Oxytocin in the amygdala facilitates maternal aggression | 077, 078 |
| Dumais, 2016 | Role of the oxytocin system in amygdala subregions in the regulation of social interest in male and female rats | 081, 082, 083, 084, 085, 086, 087, 088 |
| **First author, year** | **Title** | **Experimental group(s)** |
| Lukas, 2011 | The neuropeptide oxytocin facilitates pro-social behavior and prevents social avoidance in rats and mice | 089, 090, 091, 092 |
| Yu, 2016 | Effects of nucleus accumbens oxytocin and its antagonist on social approach behavior | 093, 094, 095, 096, 097, 098 |
| Koolhaas, 1990 | Medial amygdala and aggressive behavior: Interaction between testosterone and vasopressin | 099, 100, 101 |
| Ferguson, 2001 | Oxytocin in the Medial Amygdala is Essential for Social Recognition in the Mouse | 102, 103 |
| Lukas, 2013 | Oxytocin mediates rodent social memory within the lateral septum and the medial amygdala depending on the relevance of the social stimulus: Male juvenile versus female adult conspecifics | 104, 105, 106, 107 |
| Veenema, 2010 | Distinct correlations of vasopressin release within the lateral septum and the bed nucleus of the stria terminalis with the display of intermale aggression | 108, 109, 110, 11 |
| Beiderbeck, 2007 | Differences in intermale aggression are accompanied by opposite vasopressin release patterns within the septum in rats bred for low and high anxiety | 112, 113, 114, 115 |
| Landgraf, 2003 | Viral vector-mediated gene transfer of the vole V1a vasopressin receptor in the rat septum: Improved social discrimination and active social behaviour | 116, 117, 118, 119, 120, 121, 122 |
| Bielsky, 2005 | The V1a vasopressin receptor is necessary and sufficient for normal social recognition: A gene replacement study | 123, 124, 125, 126 |
| Engelman, 1994 | Microdialysis administration of vasopressin into the septum improves social recognition in Brattleboro rats | 127, 128, 129 |
| Everts, 1997 | Lateral septal vasopressin in rats: Role in social and object recognition? | 131 |
| Everts, 1999 | Differential modulation of lateral septal vasopressin receptor blockade in spatial learning, social recognition, and anxiety-related behaviors in rats | 132 |
| Landgraf, 1995 | VI Vasopressin Receptor Antisense Oligodeoxynucleotide into Septum Reduces Vasopressin Binding, Social Discrimination Abilities, and Anxiety-Related Behavior in Rats | 133 |
| Bosch, 2010 | Maternal behaviour is associated with vasopressin release in the medial preoptic area and bed nucleus of the stria terminalis in the rat | 134 |
| Dumais, 2016 | Involvement of the oxytocin system in the bed nucleus of the stria terminalis in the sex-specific regulation of social recognition | 135, 136, 137, 138, 139, 139, 140, 141, 142 |
| Veenema, 2012 | Vasopressin regulates social recognition in juvenile and adult rats of both sexes, but in sex- and age-specific ways | 143, 144, 145, 146, 147, 148 |
| Duque-Wilckens, 2018 | Oxytocin Receptors in the Anteromedial Bed Nucleus of the Stria Terminalis Promote Stress-Induced Social Avoidance in Female California Mice | 149, 150 |
| Dolen, 2013 | Social reward requires coordinated activity of nucleus accumbens oxytocin and serotonin | 151 |
| Bayerl, 2016 | Antagonism of V1b receptors promotes maternal motivation to retrieve pups in the MPOA and impairs pup-directed behaviour during maternal defense in the mpBNST of lactating dams | 152, 153 |
| Choleris, 2007 | Microparticle-based delivery of oxytocin receptor antisense DNA in the medial amygdala blocks social recognition in female mice | 154 |
| Lee, 2005 | Social interaction deficits caused by chronic phencyclidine administration are reversed by oxytocin | 155, 156 |
| Bosch, 2006 | Extracellular amino acid levels in the paraventricular nucleus and the central amygdala in high and low anxiety dams rats during maternal aggression: regulation by oxytocin | 157, 158, |
| Nephew, 2008 | Arginine vasopressin V1a receptor antagonist impairs maternal memory in rats | 159, 160 |
| Leroy, 2018 | A circuit from hippocampal CA2 to lateral septum disinhibits social aggression | 161, 162 |
| Rigney, 2019 | Sexually dimorphic vasopressin cells modulate social investigation and communication in sex-specific ways | 163, 164, 165 |
| Duque-Wilckens, 2016 | Inhibition of vasopressin V1a receptors in the medioventral bed nucleus of the stria terminalis has sex- and context-specific anxiogenic effects | 166, 167, 168 |
